# The Prevention Of WEight Regain in diabetes type 2 (POWER) study: the effectiveness of adding a combined psychological intervention to a very low calorie diet, design and pilot data of a randomized controlled trial

**DOI:** 10.1186/1471-2458-12-1026

**Published:** 2012-11-23

**Authors:** Kirsten AC Berk, Hanneke Buijks, Behiye Ozcan, Adriaan van’t Spijker, Jan JV Busschbach, Eric JG Sijbrands

**Affiliations:** 1 Department of Internal Medicine, division of Pharmacology, Vascular and Metabolic diseases, Erasmus Medical Centre, Rotterdam, the Netherlands; 2 Department of Medical Psychology and Psychotherapy, Erasmus Medical Centre, Rotterdam, the Netherlands

**Keywords:** Diabetes mellitus type 2, Overweight, Cognitive behaviour therapy, Very low energy diet

## Abstract

**Background:**

Obesity is of major pathogenetic importance to type 2 diabetes, it contributes to poor glycemic control and increases the risk of cardiovascular disease. Over 80% of patients with diabetes type 2 are overweight. To achieve a more favourable risk profile, changes in diet and lifestyle are needed. However, current treatment programs for obese DM type 2 patients are not effective in the long term. In this RCT, we compare the effectiveness of a Combined Psychological Intervention (CPI) and usual care in maintaining the favourable effects on weight and risk profile during 2 years of follow-up after a Very Low Calorie Diet (VLCD).

**Methods and design:**

In a randomised parallel group intervention study, 140 patients with type 2 diabetes and overweight (BMI>27 kg/m2) will be recruited from the outpatient department of the Erasmus Medical Centre.

After obtaining ≥5% of weight loss with a VLCD, participants will be randomly assigned to CPI or usual care for 10 weeks. CPI consists of cognitive behaviour therapy, problem solving therapy and proactive coping.

Primary outcome measure is weight change (kg).

Other outcome measures are Body Mass Index (BMI = weight (kg)/length (m)2), waist circumference (cm), systolic blood pressure (mmHg), HbA1c (mmol/mol), lipid levels (LDL, HDL, TG (mmol/l) and chol/HDL-ratio), antidiabetic agents and doses, cardiovascular risk profile (UKPDS), lifestyle and quality of life (EuroQol EQ-5D). Psychosocial parameters are also studied, as secondary outcomes as well as determinants for weight loss.

When successful, we want to conduct an analysis of the cost effectiveness of the intervention as compared to usual care.

**Discussion:**

We expect that a CPI after a VLCD will be effective in maintaining weight loss and improving cardiovascular risk and glycaemic control, while being cost-effective and improving quality of life in patients with type 2 diabetes.

**Clinical trials registration:**

trialregister.nl NTR2264

## Background

Diabetes has become a worldwide epidemic: the estimated global prevalence was 2,8% in 2000 and is expected to rise up to 4,4% in 2030. In the year 2000 the excess global mortality attributable to diabetes was 5,2% making diabetes the fifth leading cause of death [1]. The increase in prevalence is associated with aging of the population, the increasing prevalence of obesity in combination with physical inactivity. Cardiovascular disease is the major cause of death among patients with diabetes. An intensive pharmaceutical and behavioral therapy treatment has been estimated to reduce the risk of cardiovascular and micro vascular events by approximately 50% [2].

Approximately 80% of the people with type 2 diabetes are overweight. Losing weight is the cornerstone of prevention and treatment of type 2 diabetes: it decreases the resistance to insulin, improves glycaemic control and reduces hypertension and lipid abnormalities [3-5]. Weight loss interventions thus may contribute to a reduction of cardiovascular risk and is shown to reduce mortality in patients with type 2 diabetes and obesity [6].

Unfortunately, interventions aimed at weight reduction have only a limited effect in the long run because of regain of the initial weight loss. The required long-term lifestyle change seems difficult to achieve [7]. A meta-analysis of weight loss interventions in adults with type 2 diabetes showed that multi-component interventions including Very Low Calorie Diets may hold promise for achieving weight loss [7,8]. However, trials with long follow-up periods are lacking and the most effective type of psychological intervention remains unclear.

In the present study, we determine the effect of an integrated multi-model cognitive group therapy, in obtaining and maintaining favourable effects on weight and cardiovascular risk profile during 2 years of follow-up after a Very Low Calorie Diet.

### Very Low calorie diet

A Very Low Calorie Diet (VLCD) is a diet of less than 800 kilocalories (kcal) daily [4]. The very low intake of fat and carbohydrates, but normal amount of proteins (0,8 g/kg ideal bodyweight per day) enhances lipolysis and ketosis while preventing a negative nitrogen balance, sparing lean body mass [9].

The most commonly used VLCD’s are commercially available mixed-formula diets, containing various amounts of carbohydrate, fat and high quality protein, and have proven safety for use in patients with type 2 diabetes [9,10].

The short-term effects (i.e. < 6 months) of a VLCD in overweight patients with type 2 diabetes are favourable on weight, glycaemic control, hypertension and dyslipidaemia [11-15]. However, study outcomes are less positive in the long term (i.e. > 1 year follow-up): patients regain most of the lost weight and HbA1c returns to the same value as prior to the intervention. Nonetheless, participants often needed less anti-diabetic agents [16-18]. The study of Jazet, et al. [19] seems to be a positive exception: 18 months after a 30-day VLCD period, favourable effects on weight, blood pressure and dyslipidaemia were maintained in 18 obese patients with diabetes type 2, but with no effect on HbA1c. The authors indicated that the success was based on the strong motivation of the patients to prevent a need for insulin and a slow reintroduction of normal diet. Limitations of this study, however, were the small intervention group and the lack of a control group.

Taken together, randomized controlled trials of sufficient duration focused on prevention of weight gain after a VLCD in DM type 2 patients are required to improve the effectiveness of VLCDs.

### Weight maintenance

To achieve weight maintenance after successful weight loss, a permanent behaviour change is needed. For this purpose, a variety of psychological interventions have been implemented in weight reduction programmes. Behaviour therapy and cognitive behaviour therapy (CBT) are potential psychological interventions facilitating better maintenance of weight loss [20]. CBT is used to describe a wide range of techniques to change thinking patterns and behaviours. As a result, interventions are heterogeneous and the findings are difficult to compare.

We identified three promising psychological interventions to attain better results in sustaining weight loss: cognitive (behaviour) therapy, problem solving therapy and proactive coping. In the current study, we propose to combine them into an integrated weight maintenance programme.

### Cognitive behaviour therapy

Within cognitive psychology, humans are regarded as information processing systems, where knowledge is organized in so-called schemas. Cognitive schemas are activated by incoming information, leading to cognitions (thoughts), emotions and subsequently to behaviour. According to the founding father of the cognitive therapy, Aaron Beck, emotional disorders such as depression and anxiety disorders result from dysfunctional schemas. Cognitive therapy focuses on changing dysfunctional schemas and cognitions, using behavioral experiments and challenges [21]. In eating disorders, the cognitive model was first used to treat bulimia nervosa by adjusting overvaluation of weight and shape based on low self-esteem [22,23]. In the treatment of obesity, this model is combined with the cognitive model for addiction, which is based on the assumption that addictive behaviour is enhanced by dysfunctional cognitions during exposure to external stimuli like the smell or sight of food [24].

In a Cochrane review [20] concerning the effect of psychological interventions in the treatment of overweight and obese patients, positive effects of cognitive behaviour treatment on weight loss were described, particularly when combined with diet and/or physical activity. In a number of studies, weight loss was enhanced significantly by the addition of the cognitive component to an intervention of diet and/or exercise [25-28]. Moreover, it was found that a longer duration of the intervention and more frequent clinical contact was associated with an increased effect. However, studies with substantial follow-up (i.e. > 1 year) are lacking.

In patients with type 2 diabetes, psychotherapy (especially CBT) improves glycaemic control (HbA1c −1,0%) and psychological well-being [29]. Surprisingly, CBT did not appear to affect weight control in this patient group. Perhaps this was caused by too short duration of the studies (i.e. <6 months), as CBT may encourage long-term behavioral changes [29].

### Problem solving therapy

Problem Solving Therapy (PST) is defined as the self-directed cognitive-behavioral process by which a person attempts to identify effective or adaptive solutions for specific problems encountered in everyday living [30,31]. PST is recognized as an effective treatment of depression [32,33]. The problem-solving model for obesity treatment was first described by Perri, Nezu and Viegener in 1992 [34] and proposes that active problem solving efforts by a health care provider can help the obese person encounter everyday problems in their weight management. Perri, et al. found significantly greater long-term weight reductions in participants, who completed a PST-intervention, compared to participants receiving behavioral therapy [35]. Moreover, a recent study showed that people with better problem-solving skills lost more weight and were more compliant to therapy [36].

### Proactive coping

Proactive coping (PC), directed at an upcoming instead of an ongoing stressor, is a new focus in positive psychology research. PC consists of efforts undertaken in advance of a potentially stressful event to prevent it or modify its form before it occurs. The theory described by Aspinwall and Taylor consists of five stages: PC starts with the ‘accumulation of resources’ such as time, money, planning or organizational skills and social support, so that one is prepared as much as possible to deal with future threats. ‘Recognition’ refers to the ability to see a potential stressful event coming, followed by ‘initial appraisal’ (what is this and should I be worried about this?). The next stage consists of ‘initial coping efforts’: activities undertaken to prevent or minimize a recognized or suspected stressor. Finally, the ‘use of feedback’ involves the evaluation of the stressful event itself and the effects of one’s preliminary efforts [37].

Schwarzer and Taubert described PC as a way of aspiring a positive future by accumulating resources and realistic goal setting [38]. A recent publication has shown that the pursuit of goals was related to improved wellbeing, while preventing a negative future was not [39].

A study of the effects of PC on the self-care behaviours of newly diagnosed DM patients revealed that the treatment was highly appreciated and even after 9 months improvements in eating and exercising habits were seen. In addition, the intervention was effective in reducing both weight and blood pressure after 9 months, but had no effect on HbA1_c_ or lipid profile [40].

In the current randomized study, we compare the effect of a Combined Psychological Intervention (CPI) with usual care on weight maintenance after a Very Low Calorie Diet. Hence, we do not compare the effectiveness of Cognitive Behaviour Therapy with neither Problem Solving Therapy nor Proactive Coping, but combine these three therapies into an integrated multi-model program.

### Objectives

The primary objective of this study is to determine if an integrated multi-model cognitive group therapy, is more effective in preventing weight regain after a Very Low Calorie Diet compared to usual care.

The secondary objectives are to investigate whether an integrated multi-model cognitive group therapy following a VLCD has an effect on glycaemic control, cardiovascular risk profile, psychological variables and quality of life, and subsequently to determine which patient group benefits most of the intervention.

The tertiary objective is to determine whether the intervention is cost-effective.

### Ethical approval

This research is approved by the Medical Ethics Committee of the Erasmus Medical Centre in Rotterdam (reference number MEC-2009-143/NL26508.078.09), in compliance with the Helsinki Declaration.

## Methods/design

### Design of the study

This study is a randomized controlled parallel group intervention trial.

When patients lose equally or more than 5% of their bodyweight in the first 8 weeks of the VLCD, they are included in the intervention trial testing CPI. This arbitrary cut-off value was chosen because of its relatively large effect on the risk profile [3]. Moreover, we need a relevant weight loss to investigate weight maintenance after weight reduction. After randomization, stratified to the achieved weight loss at 8 weeks, participants are assigned to one of the following conditions:

1. VLCD + conventional treatment

2. VLCD + conventional treatment + CPI

The duration of the diet period will be 20 weeks: 8 weeks of VLCD (<800 kcal/day) followed by 12 weeks of slowly reintroducing a normal, mildly energy-restricted diet (1300 kcal/day). During week 10 of the diet, the intervention group starts with a total of 17 sessions of CPI. The first 10 sessions are weekly sessions; followed by two 2-weekly sessions, two monthly sessions, two 3-monthly sessions and 1 session with an interval of 6 months.

Outcome measurements are assessed at baseline, after finishing the intensive CPI-period at 4 months and subsequently at 1 year, 1,5 years and 2 years after randomization. At 8 weeks (randomization after VLCD), only the primary outcome (weight) will be measured.

**Figure 1:**
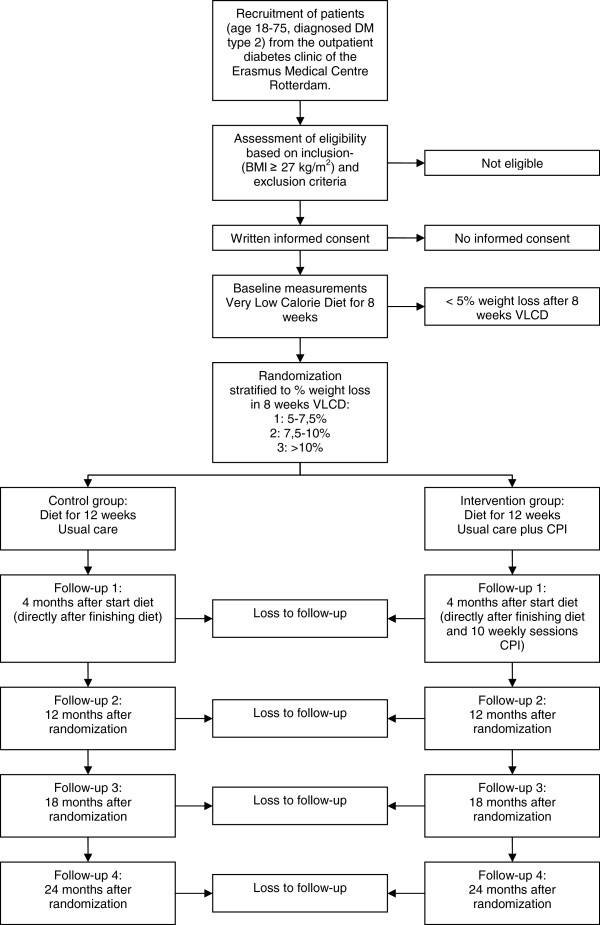
**Flow chart of participants.** VLCD = Very Low Calorie Diet; CPI = Combined Psychological Intervention.

Figure 1 shows the design of the study.

The study started in may 2010 with a pilot and inclusion will be completed in 2013. The follow-up will continue until 2015.

### Study population

Patients are recruited from the outpatient diabetes clinic of the Erasmus Medical Centre in Rotterdam by the medical team, based on the in- and exclusion criteria shown in Table 1. In our hospital, a tertiary referral centre, we see both patients with only oral antidiabetic agents and patients treated with one or more insulin doses daily. In general, our patients will have more complex disease and more comorbidity compared to the average diabetic patient referred to a GP. How ever, GP’s can also directly refer their eligible patients to our trial. Patients interested in participating in the study will receive an information letter and a questionnaire to be filled in at home. Two weeks later they will visit the outpatient clinic for an intake interview with the researcher (KACB). Eligibility will be checked again. After signing the informed consent form by the patient, the baseline measurements will be performed.

**Table 1:** Eligibility

Inclusion criteria:	
**1**	Diagnosed diabetes mellitus type 2
**2**	Age 18–75 years
**3**	BMI ≥ 27 kg/m^2^
Exclusion criteria:	
**6**	Pregnancy or lactation during the study
**7**	Inadequate expression of the Dutch language (spoken and written)
**8**	Inability to lose ≥ 5% of bodyweight during the first 8 weeks of VLCD
**9**	Severe psychiatric problems
**10**	Significant cardiac arrhythmias, unstable angina, decompensated congestive heart failure, major organ system failure, untreated hypothyroidism and/or myocardial infarction, end-stage renal disease, cerebrovascular accident or major surgery in the last 3 months.

Patients with psychiatric disorders or major psychological disturbances are excluded from the trial. Patients with eating disorders (bulimia nervosa and binge eating disorder) or depression are not excluded, since it is expected that these conditions are common among patients with type 2 diabetes and previous research has shown that cognitive behaviour therapy can have beneficial effects in these patient groups [28,41-43]. We will analyze the effect of these background variables on the outcome of the intervention.

### Randomization

Groups of 20 patients will start with a VLCD concomitantly. After 8 weeks of VLCD, the patients who lost ≥5% of bodyweight are randomly assigned to either or the intervention group (usual care + CPI) or the control group (usual care) with an allocation ratio of 1:1. The stratified randomization is computer controlled, carried out by a secretary and supervised by a statistician, who are both not involved in the trial. The reason for a stratified randomization is to avoid an unsuccessful randomization with regard to weight loss during the VLCD. It is not possible to correct afterwards in the analysis, as it is the primary outcome measurement. We defined the following strata (based on clinical experience):

1. 5% - 7,5%

2. 7,5% - 10%

3. >10% weight loss after 8 weeks of VLCD.

The statistician generates the allocation sequence and hands it over to the secretary of the department of Medical Psychology and Psychotherapy, who is not involved in the study, to guarantee allocation concealment. The secretary assigns the participants to their group and makes a list for KACB, who will plan their study appointments.

### Sample size calculation

After randomization of 75 participants, the sample size calculation was carried out by an independent statistician to base it on realistic data.

Sample size was calculated with SPSS 17.0, using the mixed-model ANOVA procedure described by Aberson [44].

Alpha was set at 0.05, power at 0.80 and the baseline-end correlation at 0.90. A clinically relevant difference between the treatment groups is 5% weight loss [3]. Mean weight at the start of the treatment is about 110 kg, with a standard deviation of 22, this equals an effect size of d = 0.25.[45] With this power calculation we estimate that we need 52 patients in each group. Anticipating a dropout rate of 25%, we aim for a total sample size of 140.

For the most important secondary outcomes we calculated the detectable difference with the sample size of n=104, alpha 0.05 and power 0.80 (Table 2). The detectable differences are quite small, so the aimed sample size seems adequate to analyse at least these secondary outcomes.

**Table 2:** Power calculation secondary outcomes

**Secondary outcome:**	**SD:**	**Detectable difference:(n = 104, β=0.8 α=0.05)**
Waist circumference	11	2.75 cm
Blood pressure sys/dias	20/20	5/5 mmHg
Total cholesterol	1.4	0.35 mmol/l
LDL cholesterol	1.0	0.25 mmol/l
HDL cholesterol	0.7	0.2 mmol/l
Triglyceride	3.0	0.75 mmol/l
HbA1c	12	3 mmol/mol
Insulin	50	12.5 IU
Depression (HADS score 0–21)	3	0.75
Anxiety (HADS score 0–21)	4	1.0
Self esteem (RSE score 10–40)	5	1.25
Fatigue (CIS score 8–56)	13	3.25
Concentration (CIS score 5–35)	8.5	2.1
Motivation (CIS score 4–28)	6	1.5
Activity (CIS score 3–21)	5	1.25
Quality of life (EuroQol 5D score 1–3)	0.5	0.125
Quality of life (EuroQol VAS 1–100)	21	5.25

### Blinding

Given the nature of the intervention, it is impossible to blind the participants, CPI-therapist and researcher (KACB). Nevertheless, we will try to keep the intervention as separate as possible from the diabetes treatment, by giving participants strict instructions not to communicate about the intervention with their medical team. Patients are given the possibility to discuss problems related to the study with an independent medical doctor. The CPI group sessions will take place in another part of the hospital, outside the diabetes clinic, to ensure that patients and medical team will not run into each other. The VLCD is not a part of the intervention and can therefore be guided by the diabetes medical team.

To avoid bias, measurements will be conducted by blinded medical assistants and analyses will be done by two analysts, independent of each other.

The allocation sequence will be revealed to the researchers once recruitment, data collection and analyses are complete.

### Interventions

#### Phase 0: very Low calorie diet

The first part of the study (before randomization) is the same for all participants and consists of a Very Low Calorie Diet (VLCD) for the duration of 8 weeks, with a phase-out of 12 weeks. When after 8 weeks of VLCD a loss of 5% or more of the initial bodyweight is reached, random allocation to the intervention- and control group will take place. Patients losing less then 5% of bodyweight in the first 8 weeks are excluded from the study and will receive usual care, including dietary advice.

The use of a VLCD is an integrated part of the dietary treatment at our outpatient diabetes clinic. Normally, the patients are free to choose between the different types of weight reduction strategies. In the present study however, only one type of weight reduction therapy is used (VLCD).

The product we use for this study is Glucerna SR®, a product specifically developed for patients with diabetes and based on a combination of slow released carbohydrates, a low-fat, high monounsaturated fatty acid content and the addition of dietary fibre. Various studies indicate that this product decreases the postprandial blood glucose levels, which may have a lowering effect on HbA1c. This diabetes-specific diet product is also used as a meal replacement for achieving weight reduction in people with type 2 diabetes, with positive effects on weight control, glycaemic control and lipid profile [46-48]. We have chosen to use a twice a day regimen of meal replacements, together with a small dinner, providing 750 kcal, 67 g carbohydrates, 54 g protein and 32 g fat (of which 16 g monounsaturated fatty acids (MUFA)) a day and RDA recommendations in micronutrients. We believe that enabling the participants to have dinner with their family during the VLCD will improve compliance.

We have developed a protocol for the use of a VLCD in patients with type 2 diabetes, where we describe the different aspects of the treatment. Oral anti-diabetic agents (except Metformin) and short-acting insulin analogues are discontinued, while the dosage of long-acting insulin analogues and biphasic mixtures is halved, to avoid severe hypoglycaemia. GLP-1 or DPP-4 inhibitors will be continued. During the VLCD and gradual transition to a normal diet, medication is adjusted by means of glucose self-control and frequent contact with diabetes nurses and dieticians. The dose of any antihypertensive medication is also adjusted during VLCD and follow-up, in consultation with the responsible physician.

Realimentation takes place by slowly reintroducing normal food in 12 weeks time, until participants use a lightly energy restricted diet (1300 kcal a day), according to national dietary guidelines. 

#### Phase 1: control group

Following the VLCD-period and gradual reintroduction of normal diet, the control group receives usual care, provided by the diabetes team of the out-patient diabetes clinic of the Erasmus Medical Centre. Usual care consists of a 3-monthly visit to the physician and diabetes nurse, including medical examinations. Dietary treatment is part of usual care by referral to a specialized dietician. Patients are free to make additional appointments with their dietician in- or outside the hospital and they can choose to follow whatever diet they would like during the follow-up period. Both the number of visits to a dietician and the dieting methods, products and duration of the diet are noted during the follow-up period of 2 years. Dieticians in our hospital are all trained in motivational interviewing to motivate the patients in changing their lifestyle. All members of the diabetic team encourage the patients to get enough exercise, but we do not refer to an exercise programme. Patients in need of psychological help (ie psychological disorder, clinical depression, major eating disorder) are referred to a clinical psychologist in- or outside the hospital. In our population, these referrals are rare. Nonetheless, the number of visits to a psychologist as well as to other caregivers will be noted during this trial. CBT or techniques of PST and PC are no part of usual care.

#### Phase 1: intervention group

After 8 weeks of VLCD, the participants allocated to the intervention group start with CPI in groups of up to 10 patients, in addition to the basic treatment for diabetes and obesity, focusing on (self) regulation of blood glucose and the prevention/reduction of complications, according to national guidelines. The CPI sessions are guided by a trained psychologist/psychotherapist, with experience in diabetes care. Two psychologists (HB and AVTS) are involved in this study, so we can investigate the ‘therapist-effect’.

The first 10 weekly sessions consist of cognitive behaviour therapy, partly based on the method developed by Werrij and colleagues from the University of Maastricht [27]. These CBT meetings are followed by 7 relapse prevention sessions.

The aim of the first 10 sessions is to restructure dysfunctional cognitions on lifestyle, weight and body perception.

Sessions 11–17 aim to prevent relapse by combining intervention techniques of CBT, PC and PST. Table 3 presents the treatment protocol in brief.

**Table 3:** Brief presentation of the treatment protocol

**Weekly sessions**	
Session 1	Introduction of the therapist and the group members
	Agreements on attendance, commitment, homework and privacy
	Setting realistic treatment goals
	Explaining the rationale of the cognitive treatment
	Introducing relevant concepts op CBT (situation, thoughts, emotions, behavior) and the use of the diary
Sessions 2 – 4	Discussing rationale and the concepts of CBT
	Explaining unrealistic and automatic thoughts
	Identifying and challenging dysfunctional cognitions about eating, weight and shape (by Socratic dialogue)
	Homework: cognitive diaries
Sessions 5 – 8	Introducing the behavioral experiment
	Challenging dysfunctional cognitions by setting up a behavioral experiment
	Identifying and challenging ‘core beliefs’, the underlying self-schemas
	Homework: cognitive diaries and behavioral experiments
Sessions 9 – 10	Preparing for the oncoming ending of weekly sessions
	Identifying and challenging dysfunctional cognitions about relapse
	Introducing relapse prevention sessions
**Relapse prevention sessions**	
Session 11	Challenging dysfunctional cognitions about relapse
	Setting goals for the long term
	Explaining Problem Solving Treatment and Proactive Coping
Session 12 – 16	Implementing PST and PC in an individual plan
Session 17	Preparing for treatment ending
	Challenging dysfunctional cognitions about treatment ending
	Personal reminder in difficult times

After session 1, the structure of each session is as follows:

– time for questions following the previous session

– discussing home-assignments

– explaining and practicing of cognitive (behavioral) techniques

– discussing new home-assignments

– summary and evaluation of the session (by completing the Session Rating Scale)

In sessions 1–5 dysfunctional cognitions about eating, weight and shape are identified and challenged. Also exercise will be included in the sessions. Cognitive diaries are introduced and participants are encouraged to use these diaries at home to record personal critical situations and dysfunctional thoughts. They also score the credibility of their dysfunctional thoughts (0-100%). Furthermore, the validity of the dysfunctional thoughts is tested and alternative thoughts are generated. Guided by examples of the group, the group is acting as a research team, investigating the validity of dysfunctional cognitions and creating alternative and more realistic thoughts. The new thoughts are again scored on credibility (0-100%). Methods used for testing the validity of a person’s thoughts are the Socratic dialogue and behavioral experiments. Behavioral experiments are set up during the session, carried out at home, and discussed in the next session.

The underlying schemas (‘core beliefs’) are explored during sessions 6–10. The validity of self schemas is tested in the same way as the other dysfunctional thoughts and recorded in the diary.

Subsequently, 7 relapse prevention meetings are held to stabilize behaviour change, with increasing intervals until the end of the study (2 years). During these relapse prevention meetings, the acquired techniques will be repeated. In addition, techniques of Problem Solving Therapy are used to help participants coping with everyday problems they encounter while implementing their lifestyle changes. Based on individually set goals, experienced problems are identified and possible solutions will be put forward in a group brainstorm session. The participant will choose the most appropriate solution and creates and carries out an implementation plan. In this plan, the participant formulates the solution as a SMART (Specific, Measurable, Attainable, Realistic, Timely) goal and considers which steps should be taken to achieve this goal. Subsequently, the participants will be encouraged to identify barriers that may arise while trying to achieve their goals. Under guidance of the psychologist, activities will be defined which can be undertaken to prevent the occurrence or diminish the effect of these potentially threats (Proactive Coping).In following relapse prevention sessions, the implementation plan is evaluated and new (sub) problems are chosen to tackle. By linking Problem Solving Therapy to Proactive Coping and cognitive restructuring of (relapse) thoughts, we hope to have created a powerful intervention to provide participants skills to prevent and cope with relapse in their behaviour.

### Outcome assessment

Outcome measurements (see below) are assessed at baseline (before start VLCD), and again at 4 months (after VLCD and 10 CPI sessions), 1 year, 1 ½ years and 2 years.

Demographic variables are assessed at baseline by use of a self-administered questionnaire, which is checked during the intake interview with the investigator.

All other secondary outcomes are assessed at baseline, after 4 months, 1 year, 1 ½ years and 2 years, using self-administered questionnaires, except of the Session Rating Scale, which is filled in after every CPI session.

Data are managed by use of the trial management system ‘EXPeRT Clinical’ of OmniComm, USA.

#### Primary outcome

The primary endpoint with respect to the efficacy of CPI is the differential course of weight (kg) between both study groups, measured at 8 weeks and at 2 years follow-up. Weight is measured to the nearest 0.1 kg, after removal of shoes using a Seca 888 compact digital flat scale.

#### Secondary outcomes

1. Anthropometric measurements: Height is measured to the nearest 0.5 cm without shoes using a Seca stadiometer. Body Mass Index is calculated as weight divided by height squared (BMI = weight (kg)/height (m)^2^). Waist circumference (cm) is measured at the level midway between the lowest rib margin and the aliac crest. Hip circumference is measured at the widest point over the buttocks. Both waist- and hip circumference are measured by the nearest 0,5 cm, using a tape-measure. Subsequently, waist-hip ratio (WHR) is calculated.

2. Cardiovascular riskprofile by use of the UKPDS risk engine [49]:

a. Systolic and diastolic blood pressure (mmHg) are measured twice in upright position, while the patient had rested for at least 5 minutes with an Omron M4-I Intelli-sense device. The second value will be used.

b. Blood samples are taken to asses total cholesterol, LDL-cholesterol, HDL-cholesterol and triglycerides (mmol/l), measured on a Roche Modular P 800, reagents used from Roche, methods used from Roche are Cholesterol Chod-pap, HDL-c plus 3^rd^ generation, LDL-c plus 2^nd^ generation and Triglycerides GPO-PAP.

3. Glycaemic control:

a. HbA_1c_ (mmol/mol), measured on a Menarini HA-8160, reversed-phase action exchange chromatography.

b. HOMA-%S calculated by measuring fasting plasma glucose (mmol/l), (measured on a Roche Modular P 800, reagent used from Roche, method used from Roche is Glucose Hexokinase) and fasting insulin (mmol/l)

c. Glucose lowering medication (insulin (IU/day) and antidiabetic agents (mg/day))

4. Psychological measurements

a. Anxiety and depression are measured by the Hospital Anxiety and Depression Scale (HADS)[50,51]. The HADS consists of a 7-item Anxiety scale and a 7-item Depression Scale. The items are scored from 0 to 3 and the range of scores is 0 – 21. A score between 0 and 7 excludes depression/anxiety. A score of 8–10 indicates a possible depression/anxiety. A score of 11–21 is indicative of a probable depression/anxiety.

b. Somatic symptoms are measured by the VOEG-13 (‘Vragenlijst Onderzoek Ervaren Gezondheid’) [52], a Dutch 13-item questionnaire used to measure the health of a population, often used in social science research. The items consist of somatic symptoms like headache, nervousness and lethargy and respondents indicate whether they have these symptoms or not. A higher score indicates more somatic symptoms and a worse perceived health.

c. Fatigue is measured by the Checklist Individual Strength (CIS) [53], which quantifies subjective fatigue and related behavioral aspects. The CIS consists of 20 statements for which the respondent has to indicate on a 7-point scale to what extent the particular statement applies to him or her (1 = Yes, that is true; to 7 = No, that is not true). The statements refer to four fatigue aspects: (1) subjective fatigue (2) reduced motivation (3) reduced activity and (4) reduced concentration. For the CIS a cut-off point of >76 has been established [54]. People with a score above this cut-off point are at an increased risk of long-term sickness absence.

d. Self-esteem is measured by the Rosenberg Self-Esteem Scale (RSE) [55]. The RSE is a 10-item questionnaire that measures global self-esteem. Items are scored on a 4-point scale. A higher score indicates a more positive self esteem. Scores below 21 indicate low self-esteem.

e. Eating disorders are measured by the Eating Disorder Examination-Questionnaire (EDE-Q) [56], a 36 item questionnaire that measures concerns about shape, weight and eating, restraint and binge eating. Subscale scores for restraint and shape, weight and eating concern range between 0–6. A higher score indicates more severe eating psychopathology. Because binge eating cannot be measured reliably by the EDE-Q, we use a questionnaire composed by Werrij, et al. [28] for diagnosing binge eating disorder (BED), based on the DSM-IV criteria for BED. BED is diagnosed when respondents report eating binges twice a week or more.

f. Session rating. How the participants value the sessions is measured by the Session Rating Scale (SRS) [57]. The SRS is an ultra brief alliance measure designed specifically for every session clinical use. The SRS consists of four 10-cm visual analogue scales (relationship scale, goals and topic scale, approach or method scale, overall evaluation scale), with instructions to place a hash mark on a line (continuum) with negative responses depicted on the left and positive responses indicated on the right. Based on a total possible score of 40, any score lower than 36 overall, or 9 on any scale, could be a source of concern and therefore prudent to invite the client to comment.

5. Lifestyle

a. We developed a lifestyle questionnaire on diet history, smoking, drinking habits, drug use and hours sleep. (Additional file 1)

b. Physical activity is measured using the SQUASH (Short Questionnaire to Assess Health Enhancing Physical Activity) [58]. The SQUASH collects days per week, average time per day, and effort for physical activities such as commuting activities, leisure time and sport activities, household activities, and activities at work or school. Total minutes of activity are calculated for each question by multiplying frequency (days per week) by duration (minutes per day). Activity scores for separate questions are calculated by multiplying total minutes of activity by an intensity score (range 1–9). The total activity score is calculated by taking the sum of the activity scores for the separate questions.

6. Cost-effectiveness

a. Quality of life is measured by the EuroQol (EQ-5D) [59,60]. The EQ-5D is 5-item self report questionnaire on which participants report if they experience any problems in mobility, self-care, usual activities, pain and anxiety/depression. Each dimension has 3 levels: no problems, some problems and severe problems. The scores on the three dimensions can be combined into one co called ‘utility’ score, which represents the societal value of quality of life. The utility score has a range from 1.00 (the value of health without health problems) till 0.00 (the value of health problems as bad a death). This societal value of quality of life is used as input for so called Quality Adjusted Life Years (QALY) analysis, as the societal perspective is the preferred perspective in health economics. The EuroQol instrument contains also the EQ-VAS, a vertical visual analogue scale with the anchors best imaginable health (score of 100) and worst imaginable health (score of 0). This EQ-VAS represents the patient’s perspective of quality of life.

b. Costs are measured by the Trimbos/iMTA Questionnaire for Costs associated with Psychiatric Illness (TiC-P) [61]. The TiC-P measures direct medical costs due to healthcare utilization during the past four weeks. Also, it registers the indirect non-medical costs due to productivity loss during the past two weeks.

### Patient follow-up and compliance

Follow-up measurements will take place combined with evaluation group meetings.

For patients in both groups, follow-up visits to the physician, diabetes nurse and dietician are registered.

A common limitation of weight loss studies is a selective loss to follow-up: higher drop-out rates occur among patients, who do not achieve their weight loss goals. We want to tackle this problem by use of incentives to keep all patients in the trial: motivating phone calls of the researcher, little gifts during the program, etc.

Further more, we will perform a follow-up and analysis of the drop-outs after completion of the study and compare completers and drop-outs at base-line. For this purpose, the drop-outs will be interviewed by a medical student, who is not involved in the study.

Compliance to the intervention is assessed by registration of the attendance to the CPI sessions: participants are considered non-compliant when they are absent on more then 8 sessions. Since we expect that the treatment will be less effective when more then 8 sessions are missed, this non-compliant participants will be considered drop-outs.

We have conducted a pilot study to improve our research protocol. We will not perform interim analyses and we will not define stopping rules, since the intervention has no serious side-effects.

### Data/results pilot study

In order to test the protocol we conducted a pilot study, in which we included 13 patients to asses the study design and logistic pathways of the VLCD-period and the first 10 weeks of CPI. In this pilot study, we found that the protocol was feasible and that no major changes had to be done. The only change we made was in the lay-out of the questionnaires, making them better understandable to our participants. Results of this pilot study (baseline and T=4 months) are shown below (Table 4 and 5).

**Table 4:** baseline characteristics pilot group

	**Baseline (n=13)**	
**Sex (%)**	Males	25%
	Females	75%
**Age (y)**		49.5
**Insulin dependant (%)**	Insulin dependant	75%
	Non-insulin dependant (OAD)	25%

**Table 5:** Outcome measurements pilot group

	**Baseline (n=13)**	**T1 = 4 months (n=13)**	**95%CI**
**Weight (kg)**	114.8	106.0***	[5.2-12.4]
**Waist circumference (cm)**	121.4	113.7**	[3.2-12.2]
**Systolic BP (mmHg)**	144.6	127.9*	[1.1-32.2]
**Diastolic BP (mmHg)**	86.1	78.0	[−0.4-16.7]
**Total cholesterol (mmol/l)**	5.0	4.5	[−0.2-1.3]
**HDL cholesterol (mmol/l)**	1.7	1.3	[−0.3-1.3]
**LDL cholesterol (mmol/l)**	2.6	2.5	[−0.3-0.5]
**Triglycerides (mmol/l)**	3.6	2.6	[−1.1-3.0]
**HbA1c (mmol/mol)**	66.3	66.6	[−9.8-9.1]
**Insulin (IU)**	82.3	32.3*	[7.3-94.7]
**Depression score (HAD)**	5.7	4.6	[−1.5-3.7]
**>8 = (sub)clinical**			

Paired samples *T*-test *p<0.05 **p<0.01 ***p<0.001.

### Statistical analyses

All analyses will be conducted according to the intention-to-treat as well as the on-treatment principle. We consider participants on-treatment when they miss less then 8 sessions CPI.

At baseline, comparability between the control- and intervention group will be assessed to test the success of the randomization. As measures of central tendency for numerical data we will use the mean (in case of normal distribution) and median values (in case of non-normal distribution), with respectively the standard deviation and interquartile range as measures of dispersion.

Mixed modelling, also known as random effect modelling, multilevel or hierarchical linear regression analyses will be applied for longitudinal analyses of the data. Mixed modelling can efficiently handle data with missing and unbalanced time-points. It corrects for bias when absence of data is dependent on characteristics that are present in the models (missing at random, MAR) [62]. There will be two levels in the models. The patients constitute the upper level, their repeated measures the lower level. First, for each outcome variable a saturated model will be postulated, with the primary or secondary outcomes as dependent variables. The saturated models will include treatment group, time, quadratic time, logarithm of time and all treatment-time interactions as fixed effects. The deviance statistic [63] using restricted maximum likelihood [64] will be applied to determine the covariance structure. Next, using Wald tests, the saturated models will be reduced by eliminating insignificant fixed effects. The significance of the difference between the saturated models and the parsimonious final models will be determined with the deviance statistics using ordinary likelihood.

## Discussion

To our knowledge, this trial is the first randomized controlled trial to test the effects of an integrated multi-model cognitive group therapy in the battle against weight regain after a successful weight loss intervention (VLCD) in patients with type 2 diabetes. Weight regain is common in the obese population in general, but even more pronounced in the diabetes type 2 population, and with more devastating effects on their health outcomes. The strength of this study is the combined psychological intervention (CPI) of cognitive restructuring, problem solving treatment and proactive coping, which will provide powerful tools to the participants for maintaining behavioral change and improving health outcomes. We expect that adding CPI to a VLCD will be effective in maintaining weight loss, improving lifestyle and, as a result, leading to improved glycaemic control and a reduction of cardiovascular risk.

We expect that a number of factors may influence the treatment effect, therefore we will also examine potential determinants of weight maintenance, such as depression, anxiety, self-esteem, fatigue, somatic symptoms, eating disorders and lifestyle. Apart from being predictive for the success of the intervention, these factors may be changed by the treatment itself and therefore will be followed over time as secondary outcome measurements.

It is expected that if the intervention is successful, it will also be cost effective since the costs of group counselling are relatively low and the expected reduction of medication, prevention or delay of complications and a reduction of hospital admissions are considered major cost savings. If the therapy is indeed effective, a formal cost effectiveness analysis will be performed.

Few studies have implemented interventions to sustain weight loss for longer then 18 months. In this study, we opt for a follow-up period of 2 years, enabling predictions on long-term behavioral change and weight maintenance. During the follow-up period, participants will have relapse-prevention group meetings with increasing intervals to stabilize behaviour. It is known that the effect of an intervention reduces when the contact with the therapist stops, and one can therefore expect that the participants experience a relapse in their behaviour after the follow-up period. The last session op CPI will be at 1,5 years after randomization, leaving the participants 6 months ‘to themselves’. Obviously, our patients remain under supervision of the healthcare team after completion of the study and will be encouraged to continue their good habits. Nevertheless, after 5 years, we will try to repeat the assessment of the main outcome variables to determine the long-term effect.

### Limitations

This trial is explanatory in design in relation to the measures of process but pragmatic in terms of the comparison with usual care rather than a specified alternative group. We chose this design to enable analysis of an additional intervention on top of usual care in a population already receiving multiple interventions. The pragmatic approach and planned economic analysis aim to facilitate implementation of the intervention when successful, but this design does not identify the optimal psychological therapy.

If the intervention is successful, it cannot be ascertained which of the 3 therapies involved determined the success. However, each of the treatments has been studied separately and is more or less proven effective in achieving weight reduction and maintenance [20,26,28-32,34-36,40]. We expect that a diet with a combination of several forms of cognitive behaviour therapy together will have a greater impact than the individual factors.

Clearly the assumption is that the overlap of effects of the different types of interventions is negligible. This is not necessarily true. Hence, the present study is designed to study the effect of a combination therapy, but cannot identify the optimal combination of interventions.

Another limitation is the comparison to usual care only. One could argue that the participants in the intervention group may benefit from the attention they get, and not necessarily from the CPI. In our study design, it is impossible to distinguish between the effect of attention and the effect of the intervention itself. Our choice to compare our intervention on top of usual care with usual care only, is based on the fact that this kind of comparative effectiveness design has more clinical relevance. Such design is a necessary condition for a cost effectiveness analysis, as cost effectiveness is measured in relation to the dominant alternative treatment strategy. Moreover, the individual interventions have been shown effective in comparison with other psychological interventions, exercise or placebo as a control [27,35,40,65].

We are aware of the fact that our population is not generalizable to the entire group of overweight patients with type 2 diabetes.

One can speculate that patients, who agree to participate in the study and are able to achieve a 5% weight reduction in 8 weeks, are expected to be more motivated then patients, who refuse to participate or who fail to lose sufficient weight. Randomization will distribute this selection bias equally to the CPI and the control group. Nonetheless, our findings will solely be applicable to patients, who are motivated to reduce their body weight.

Our hospital is a tertiary referral centre and as a result the patients often have end stage disease with multiple complications. Due to this selection, motivation, compliance and treatment outcome are expected to be low, potentially resulting in underestimation of the effect. However, GP’s will also be able to refer their eligible patients directly to the trial. Those patients often have less severe disease and complications.

Insufficient command of the Dutch language is an exclusion criterion of this investigation, because the cognitive training is given in Dutch and the workbook and homework assignments must be understood and carried out in Dutch as well. In this way, a part of the immigrant population is excluded and the study population will not reflect the entire diabetic population of the Erasmus MC.

We will use incentives (motivating phone calls, little booklets etc.) to minimize drop-out. This effect will be the same for the control group and the intervention group, not affecting the results. However, it will have an effect on the generalizability of the study because the less motivated participants are more likely to remain in the study where they would normally drop-out.

We are aware of the impact, especially on the control group, of the large measurement burden due to all the psychological questionnaires. We will discuss this with the participants beforehand and expect that they find 5 times 30 minutes of completing questionnaires is acceptable in the light of the weight loss intervention they will receive.

### Future implementation

If this intervention proves to be effective and cost-effective, we will promote implementation into diabetes care.

In the present study, we use a VLCD because it leads to quick and substantial weight loss, but off course the multi-model cognitive therapy can be combined with any weight loss intervention (i.e. low calorie diet, low carbohydrate diet, exercise), to sustain the effect.

The study will start in 2010 and the inclusion of patients will take approximately 3 years. After the follow-up period of 2 years, we expect the results to become available in 2015.

## Abbreviations

BMI: Body Mass Index; CBT: Cognitive Behaviour Therapy; PST: Problem Solving Therapy; PC: Proactive coping; CPI: Combined Psychological Intervention; VLCD: Very Low Calorie Diet; LDL: Low Density Lipoprotein; HDL: High Density Lipoprotein.

## Competing interests

The authors declare that they have no competing interests. To be more specific: there is no financial or other relationship between the authors and the manufacturer of the used product Glucerna SR. This trial is investigator driven and has received no external funding.

## Authors’ contributions

KACB participated in the design of the study and wrote the first draft of the manuscript. HB participated in the design of the psychological intervention and helped to draft the manuscript. EJGS, AS, JJVB and BO participated in the design and coordination of the study and revised the manuscript critically. All authors read and approved the final manuscript.

## Pre-publication history

The pre-publication history for this paper can be accessed here:

http://www.biomedcentral.com/1471-2458/12/1026/prepub

## Supplementary Material

Additional file 1 Lifestyle questionnaire. Click here for file
