# Supplementary material for: The Prevention Of WEight Regain in diabetes type 2 (POWER) study: the effectiveness of adding a combined psychological intervention to a very low calorie diet, design and pilot data of a randomized controlled trial
Source: BMC Public Health. 2012 Nov 23;12:1026. doi: 10.1186/1471-2458-12-1026 (PMC3599599; doi:10.1186/1471-2458-12-1026)
Supplement: Additional file 1 — Lifestyle questionnaire. [file 1471-2458-12-1026-S1.pdf]

Lifestyle questionnaire

Research number

1. General characteristics

Date (day / month / year)

What is your gender? *Please circle your answer* — male / female

What is your date of birth? (day / month / year)

What is your highest completed education?
Please tick one answer

- Basisschool (lager onderwijs, speciaal onderwijs)
- Lager beroepsonderwijs (bijv. LTS, LHNO, LEAO, huishoudschool)
- Middelbaar algemeen onderwijs (bijv. VMBO, ULO, MULO, MAVO)
- Middelbaar beroepsonderwijs (bijv. MTS, MEAO, MHNO)
- Voortgezet algemeen onderwijs (bijv. HBS, HAVO, VWO, gymnasium)
- Hoger beroepsonderwijs (bijv. HTS, HEAO, HBO, HHNO)
- Wetenschappelijk onderwijs (universitaire opleiding)
- other, namely:

What is your marital status?
Please tick one answer

- Maried
- Unmaried
- Divorced
- Widow(er)

What is your country of origin?

- Netherlands
- Morocco
- Antilles
- Suriname
- Turkey
- Other, namely:

What is your current occupation?

Do you make use of social benefits?

- No
- AOW
- Bijstand
- PGB
- Studiefinanciering
- WAO/WIA
- WW
- Ziektewet

To maintain contact we would like you to provide your contact details.

Telephone number:
Emailaddress:

2. Your health

	year
In what year the diabetes is diagnosed?	

	yes	no
Do you have physical symptoms of your diabetes		

If so, which?
Only mention complaints which are caused by your diabetes

fatigue	
headache	
thirst	
excessive urination	
itch	
severe hypoglycemia	
severe hyperglycemia	
foot complaints	
eye complaints	

What medicine do you use for your diabetes?

Insulin: *Dosage in units*

	yes		morning	midday	evening	bedtime
Insulatard						
Lantus						
Levemir						
Novorapid						
Humalog						
Apidra						
Inshuman Rapid						
Actrapid						
Novomix 30						
Insulinepump		units per day:				
Other, namely:						

Other antidiabetic agents: *Dosage in mg:*

	yes	morning	midday	evening	bedtime
Metformine					
Glimepiride (Amaryl)					
Gliclazide (Diamicron)					
Tolbutamide (Rastinon)					
Glibenclamide (Daonil)					
Sitagliptine (Januvia)					
Exenatide (Byetta)					
Other, namely:					

	yes	no
Do you experience hypoglycemia?		

What frequency?

1 or more times per year	
1 or more times per month	
1 or more times per week	
1 or more times per day	

Experience hypo bij glucose value of...

> 4	mmol/l	
3,6	mmol/l	
3,3	mmol/l	

3 mmol/l	
2,7 mmol/l	
< 2,5 mmol/l	

How may times did you need the help of others in treating a hypoglycemia last year, because you weren't able to react yourself?

Never	
1-2 times per year	
every other month	
once per month	
more often	

Do you use any other medication?

yes	no

If yes, which?
for example antihypertensives, lipid lowering medication, etc

Medication:	*Dosage:* morning	midday	evening	bedtime

Would you be so kind to bring all your medication to the research appointment?

Do you consider yourself religious?

yes	no

If so, what religion?

Buddhism	
Christianity	
Hinduism	
Islam	
Judaism	
Other, namely:	

Does your religion come with nutritional guidelines?

yes	no

If yes, mention below what kind of nutritional guidelines:

Do you currently follow a diet?

yes	no

If yes, which diet?

Diabetesdiet	
weight loss diet	
diet for lowering cholesterol	
diet for lowering high blood pressure	

other diet, namely: [ ]

Do you have a history of weight loss dieting? yes / no

If so, which diet?

Low energy diet	yes	no
Meal replacements (Modifast, Slimfast, Cambridge, Herbalife, ed)	yes	no
Weight loss programme (Weight Watchers, New Figure, Scarsdale, ed)	yes	no
self help book (Sonja Bakker, Margriet puntendieet, ed)	yes	no
Other, namely:	yes	no

[ ]

Have you consulted a dietitian over the last year? yes / no

If yes, how many times? [ ] times

Do you have a history of eating disorders? yes / no

If yes, which?

Anorexia nervosa	yes	no
Bulimia nervosa	yes	no
Binge eating disorder	yes	no

If so, have you been treated for your eating disorder? yes / no

3. Lifestyle habits

Do you smoke? yes / no

If yes, what do you smoke?	*amount:*	
Cigarettes		per day
Low in nicotine		per day
Cigars		per day
Pipe		per day

Do you use alcohol? yes / no

If yes, how many glasses of beer per week?		glasses per week
If yes, how many glasses of wine per week?		glasses per week
If yes, how many glasses of liquor per week?		glasses per week
If yes, how many glasses of mixed drinks per week?		glasses per week

Do you use (soft)drugs? yes / no

If yes, how many times per week?

Softdrugs		times per week
harddrugs		times per week

How many hours of sleep do you get per night on average? [ ] hours per night
